# Antioxidant Activity with Increased Endogenous Levels of Vitamin C, E and A Following Dietary Supplementation with a Combination of Glutathione and Resveratrol Precursors

**DOI:** 10.3390/nu12113224

**Published:** 2020-10-22

**Authors:** Priscilla Biswas, Cinzia Dellanoce, Alessandra Vezzoli, Simona Mrakic-Sposta, Mauro Malnati, Alberto Beretta, Roberto Accinni

**Affiliations:** 1 SoLongevity Research, 20121 Milan, Italy; alberto.beretta@solongevity.com (A.B.); roberto.accinni@gmail.com (R.A.); 2 Institute of Clinical Physiology, National Council of Research (IFC-CNR), ASST Grande Ospedale Metropolitano Niguarda, 20162 Milan, Italy; cinzia.dellanoce@ospedaleniguarda.it (C.D.); alessandra.vezzoli@cnr.it (A.V.); simona.mrakicsposta@cnr.it (S.M.-S.); 3 Unit of Viral Evolution and Transmission, IRCCS Ospedale San Raffaele, 20132 Milan, Italy; mauro.malnati@hsr.it

**Keywords:** glutathione, polydatin, precursors, vitamins, supplements, antioxidant, anti-inflammatory, sirtuins, aging, lifespan

## Abstract

The effects of two different dietary supplements on the redox status of healthy human participants were evaluated. The first supplement (GluS, Glutathione Synthesis) contains the precursors for the endogenous synthesis of glutathione and the second (GluReS, Glutathione and Resveratrol Synthesis) contains in addition polydatin, a precursor of resveratrol. To assess the influence of GluS and GluReS on the redox status, ten thiol species and three vitamins were measured before (t0) and after 8 weeks (t1) of dietary supplementation. An inflammatory marker, neopterin, was also assessed at the same time points. Both supplements were highly effective in improving the redox status by significantly increasing the reduced-glutathione (GSH) content and other reduced thiol species while significantly decreasing the oxidized species. The positive outcome of the redox status was most significant in the GluRes treatment group which also experienced a significant reduction in neopterin levels. Of note, the endogenous levels of vitamins C, E and A were significantly increased in both treatment groups, with best results in the GluReS group. While both dietary supplements significantly contributed to recognized antioxidant and anti-inflammatory outcomes, the effects of GluReS, the combination of glutathione and resveratrol precursors, were more pronounced. Thus, dietary supplementation with GluReS may represent a valuable strategy for maintaining a competent immune status and a healthy lifespan.

## 1. Introduction

Oxidation [1] and inflammation [2] are key physiologic processes that are at the basis of several chronic diseases. Aging [3] and external factors contribute to oxidative stress, which is an exacerbation of oxidative reactions that cause damage to DNA [4], lipids [5] and proteins/enzymes [6]. A link between oxidation and inflammation exists, with antioxidants having been shown to help to prevent excessive inflammatory responses [7].

Several antioxidant factors [8] are continuously activated to delay the age-related pro-oxidizing shift in the redox state. Glutathione represents one of the most important and ubiquitous among those factors [9,10]. Glutathione is a thiol tripeptide formed by glutamate (Glu), cysteine (Cys) and glycine (Gly) which is synthesized in the cytosol of all mammalian cells [11] where it reaches a very high concentration, in the mM range [10] and is actively transported in the inter-membrane space of mitochondria [12]. Most of glutathione synthesis occurs in the liver where it is subsequently secreted and transported to other organs, to assist in maintaining inter-organ glutathione homeostasis. Glutathione is also the principal mediator of detoxification reactions for protection from xenobiotic insults (drugs, toxins, etc.) [10].

The levels of glutathione decline with age [13,14,15] and their replenishment through dietary supplementation represents a potential strategy to achieve a healthy aging [16]. However, the majority of exogenously ingested glutathione is destroyed within the gastrointestinal tract. To overcome this limitation, different approaches have been attempted, including delivering glutathione by liposomes [17]. Thus far, the most effective approach has been shown to be the induction of endogenous synthesis of glutathione through dietary supplementation of its precursors [18].

This study was designed to test, in healthy human participants, the antioxidant and anti-inflammatory efficacy of two dietary supplements containing either the glutathione precursors glutamine, α-ketoglutarate and alanine [19,20], *N*-acetylcysteine [21,22] and glycine [23] alone (GluS), or combined with polydatin (GluReS), which acts as a precursor of resveratrol [24]. In order to provide a complete picture of the redox status before and after dietary supplementation, the antioxidant efficacy was evaluated by measuring ten thiol species and three endogenous vitamins. The anti-inflammatory effects were investigated by measuring the inflammatory marker neopterin [25].

Resveratrol (RV) is a well-known polyphenol which has gained wide attention for its anti-aging activity in animal models [26] and its observed ability to counteract the onset of many age-related comorbidities in humans [27], mainly through the activation of a family of deacetylating enzymes, the sirtuins [28,29]; RV however, has poor bioavailability. Polydatin (PD), which differs from RV only for a glycosidic residue, is endowed with a higher solubility that permits uptake by glucose receptors into the cells [30] where RV is then released via enzymatic hydrolysis. In addition, PD is more resistant to oxidation than RV [31] and also presents a higher free radical scavenging activity compared to RV [32]. Of interest, red wine contains greater amounts of PD than RV [33,34]. Similarly to RV, PD has been shown to activate sirtuins in a number of animal models [35,36,37,38]. In turn sirtuins are able to trigger phase II enzymes (anti-oxidant) [39] and enhance the glutathione system [40] activating the main enzymes involved in glutathione metabolism: GSH-Peroxidase (GPX) [41] and GSH-S-Transferase (GST) [42]. GPX and GST are potent mediators of the anti-oxidant and detoxifying function of GSH. Therefore, we hypothesized that the addition of PD to GSH precursors should boost the anti-inflammatory action and potentiate the anti-oxidant system, possibly leading to a synergistic effect. The antioxidant efficacy of the two dietary supplements GluS and GluReS was evaluated by the assessment of thiols redox status in erythrocytes, adopted as cellular model, and in plasma.

## 2. Materials and Methods

### 2.1. Study Design

To evaluate the anti-inflammatory and antioxidant effects of a supplement of glutathione precursors, with and without PD, we conducted a randomized clinical trial.

The healthy participants underwent a medical evaluation to determine his/her eligibility. The exclusion criteria were: (1) pregnancy; (2) regular use of drugs which inhibit the metabolism of homocysteine (such as methotrexate, anti-epileptic drugs); (3) serious disease; (4) chemotherapy. Each participant signed an informed consent.

Thirty adult men and women aged 45–75 years were randomly assigned to one of two treatment groups by adaptive minimization, balancing for age, gender and smoking status. The treatment groups received either GluReS (glutathione precursors + PD) or GluS (glutathione precursors only). Participants were blinded to the treatment (bottles were labeled A or B and capsules of GluReS and GluS were undistinguishable). Each treatment group was composed of 15 participants: a first group of seven males and eight females, mean age: 59 years ± 12 SD, who received GluReS (glutathione precursors + PD) and a second group of eight males and seven females, mean age: 60 years ± 10 SD who received GluS (glutathione precursors only). Five and four smokers were present in GluReS and GluS group, respectively. Anthropometric data of the two groups are reported in Table 1.

Both dietary supplements were supplied as capsules, 2 capsules/day to be taken on a full stomach (each capsule after a meal).

The formulation of the two dietary supplements is shown below in Table 2.

Both dietary supplements were produced by Solimè Srl, (Milan, Italy) a company certified according to UNI EN ISO 9001:2015, which guarantees that the productions are carried out in a HACCP self-control system and in compliance with Good Manufacturing Practices (GMP) according to ISO 22716 and the indications of the regulations in force.

The participants did not interrupt consumption of usual drugs prescribed by their doctors and were asked to report adverse events or concomitant medications occurring during the study period. The participants were also asked to notify any changes in lifestyle or dietary habits.

The study period was eight weeks and each participant underwent a blood and urine sampling at the beginning (t0) and at the end (t1) of the study. Biological samples were obtained in the morning after an overnight fast. Approximately 5 mL of blood were drawn from the antecubital vein. The blood samples were collected in ethylenediaminetetraacetic acid dipotassium salt (K_2_EDTA) vacutainer tubes (Becton Dickinson Company, Oxford UK), and blood was separated by centrifuge (5702R, Eppendorf, Germany) at 3000× *g* for 5 min at 4 °C. Samples of plasma and erythrocytes were then immediately stored in multiple aliquots at −80 °C until the analyses. Aliquots of the urine were stored at −20 °C until the analyses were performed.

This study was conducted in accordance with the Good Clinical Practice guidelines and the Declaration of Helsinki. Study approval was received from the IRCCS Ospedale San Raffaele (Milan, Italy) ethical committee.

### 2.2. High Performance Liquid Chromatography (HPLC) Determination of the Principal Circulating Thiols in Plasma and Erythrocytes

Total and reduced thiols were measured in plasma and erythrocytes following a validated method previously published, using two different procedures for preparation of samples [43]. In brief, two aliquots of each sample were prepared, one for the total and one for the reduced thiols. The latter was treated with Tris-(2-carboxyethyl)-phosphine hydrochloride (TCEP) as reducing agent, then both aliquots underwent a precipitation step with 10% trichloroacetic acid (TCA), followed by centrifugation at 14,000× *g* for 10 min at 4 °C. Clear supernatant (100 μL) was incubated 90 min at room temperature with 4-fluoro-7-sulfamoylbenzofurazan (ABD-F, derivatizing agent) before chromatographic analysis. Thiols’ separation was performed at room temperature through high pressure isocratic liquid chromatography on a Discovery analytical column (250 × 4.6 mm, Supelco, Sigma-Aldrich, Bellefonte, PA 16823-0048, USA) eluted with 0.1 M acetate buffer, pH 4.0–methanol, 81:19 (v/v) with a flow rate of 1.0 mL/min. Fluorescence intensity was measured by a Jasco fluorescent spectrophotometer with excitation and emission wavelengths of 390 and 510 nm, respectively. Sample concentration was obtained using a standard calibration curve. The units of measurement are µM. The concentration of oxidized forms was calculated as the difference between total and reduced thiols forms. Oxidized glutathione forms encompass the dimers (GSSG), the mixed disulfide forms (GSSR) and the protein mixed disulfide forms (ProSSG) [44].

### 2.3. HPLC Determination of Vitamin C (ascorbic acid), Vitamin A (retinol), Vitamin E (α-tocopherol)

HPLC determination of the three vitamins were performed using commercially available kits with European certification from Chromsystems Instruments and Chemicals GmbH (Grafelfing, Germany): for vitamin A and E order n. 34000, for vitamin C order n. 65065. Reproducibility: intra-assay for vitamin A coefficient of variation (CV) is 2.5%, for vitamin E CV is 2.4%, for vitamin C CV is 3.8%; inter-assay for vitamin A CV is 5.0%, for vitamin E CV is 4.9%, for vitamin C CV is 4.8%. Recovery for vitamin A is 106%, for vitamin E is 101%, for vitamin C is 97–103%. Linearity for vitamin A is 0.02–5.0 mg/L, for vitamin E is 0.25–45.0 mg/L, for vitamin C 0.4–100 mg/L. Limit of quantification (LLOQ) for vitamin A is 0.02 mg/L, for vitamin E is 0.25 mg/L, for vitamin C is 0.40 mg/L. Vitamin E concentrations (µM) were normalized to total cholesterol (mM) for a more accurate evaluation [45].

### 2.4. HPLC Determination of Urinary Neopterin

Urinary neopterin concentrations were measured by an isocratic HPLC method and were normalized to urine creatinine concentrations. Urine samples, stored at −20 °C were thawed and centrifuged at 13,000 rpm for 5 min at 4 °C; the supernatant was then adequately diluted with chromatographic mobile phase (15 mM of K_2_HPO_4_, pH 3.0). Neopterin and creatinine levels were measured using a Varian instrument (pump 240, autosampler ProStar 410) coupled to a fluorimetric detector (JASCO FP-1520, λ_ex_ = 355 nm and at λ_em_ = 450 nm) for neopterin detection and to an ultraviolet-visible detector (Shimadzu SPD 10-AV, λ = 240 nm) for creatinine determinations. Analytic separations were performed at 50 °C on a 5 µm Discovery C18 analytical column (250 × 4.6 mm I.D., Supelco, Sigma-Aldrich, Bellefonte, PA 16823-0048, USA) at flow rate of 0.9 mL/min. The calibration curves were linear over the range of 0.125^−1^ μmol/L and of 1.25^−10^ mmol/L for neopterin and creatinine levels, respectively. Inter-assay and intra-assay coefficients of variation were <5%.

### 2.5. Statistics

Data are expressed as mean ± SD and were analyzed using parametric statistics following mathematical confirmation of a normal distribution using Shapiro-Wilks W test. Experimental data were compared by ANOVA variance analysis followed by Tukey’s multiple comparison test to further check the among group significance. Statistical analyses were performed using the software Prism 8 (GraphPad Prism 8.3, Software, Inc., San Diego, CA, USA). A *p*-value of <0.05 was considered statistically significant. Change ∆% estimation [(post value-pre value)/pre value) * 100] was used to compare the changes that occurred in the two study groups after the 8 weeks of diet supplementation and it is also reported in the text. The prospective calculation of the sample size was determined choosing the value of reduced glutathione [46]. At a power of 80% the number of participants of 13 was calculated, which is below the number of participants recruited for this study.

## 3. Results

All participants completed the eight weeks (t1) of dietary supplementation and no adverse effects were reported. Furthermore, no significant differences between the two groups examined at baseline were found.

**Figure 1:**
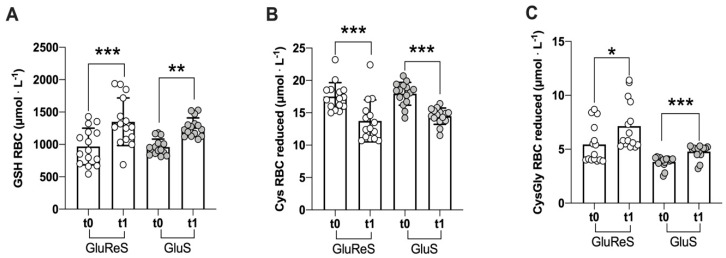
Scatter dot plot of reduced thiol species measured in erythrocytes before (t0) and after 8 weeks (t1) of dietary supplementation with GluReS (Glutathione and Resveratrol Synthesis) or GluS (Glutathione Synthesis). (**A**) reduced glutathione (GSH), (**B**) reduced cysteine (Cys), (**C**) reduced cysteinylglycine (CysGly); RBC = red blood cells. Data are expressed as the mean ± SD. Changes over time (t1 vs. t0) were significant at: * *p* < 0.05; ** *p* < 0.01; *** *p* < 0.001.

The level of endogenous reduced thiols in erythrocytes at t0 and t1 are shown in Figure 1. A significant increase of reduced glutathione (GSH) was induced by both dietary supplements (*p* < 0.001 for GluRes and *p* < 0.01 for GluS) (Figure 1A); however, the increase was greater in GluReS compared to the GluS group (+40% and +32%, respectively). Cysteine (Cys) instead (Figure 1B) decreased significantly (*p* < 0.001) in both groups, as expected because it was efficiently used to produce GSH in the erythrocytes. The decrease was superior in group GluReS versus group GluS (−22% and −19%, respectively). Finally, a catabolite of GSH, cysteinylglycine (CysGly) was also measured (Figure 1C); a significant (*p* < 0.05 vs. *p* < 0.001) increase in both groups was found, and also in this case the increase was higher in group GluReS compared to group GluS (+32% versus +25%).

**Figure 2:**
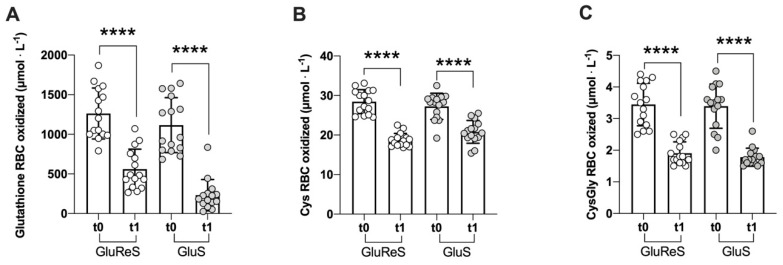
Scatter dot plot of oxidized thiol species measured in erythrocytes before (t0) and after eight weeks (t1) of dietary supplementation with GluReS or GluS. (**A**) oxidized glutathione, (**B**) cysteine (Cys), (**C**) cysteinylglycine (CysGly); RBC = red blood cells. Data are expressed as the mean ± SD. Change over time (t1 vs. t0) was significant at: **** *p* < 0.0001.

The results of the oxidized thiols in erythrocytes are depicted in Figure 2. A highly significant (*p* < 0.0001) decrease from t0 to t1 was observed in: oxidized glutathione (−56% vs. −79%, Figure 2A); oxidized Cys (−34% vs. −24%, Figure 2B) and oxidized CysGly (−44% vs. −47%, Figure 2C) for group GluReS compared to group GluS, respectively. The oxidized glutathione (Figure 2A) represents a total pool including not only the dimers (GSSG), but also the mixed disulfide (GSSR) and the protein mixed disulfide forms (ProSSG).

**Figure 3:**
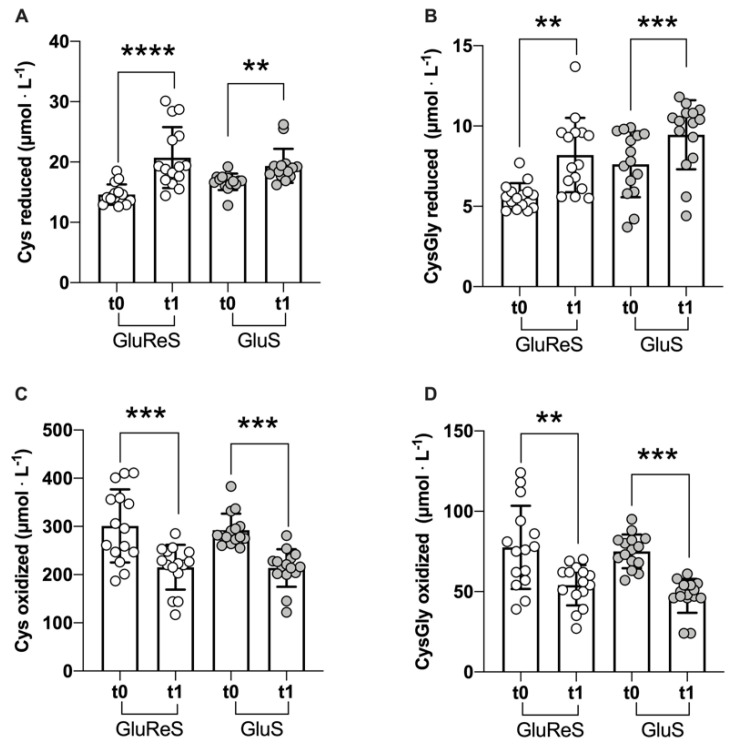
Scatter dot plot of reduced and oxidized thiol species measured in plasma before (t0) and after 8 weeks (t1) of dietary supplementation with GluReS or GluS. (**A**) reduced cysteine (Cys), (**B**) reduced cysteinylglycine (CysGly), (**C**) oxidized cysteine (Cys), (**D**) oxidized cysteinylglycine (CysGly). Data are expressed as the mean ± SD. Changes over time (t1 vs. t0) were significant at: ** *p* < 0.01; *** *p* < 0.001; **** *p* < 0.0001.

The reduced and oxidized thiols were evaluated also in plasma, except for glutathione due to its almost undetectable levels in plasma [9]. Figure 3 summarizes the data obtained at t0 and t1 for both treatment groups. Reduced Cys increased highly significantly (*p* < 0.0001) in GluReS group and significantly (*p* < 0.01) in the GluS group (+42%, vs. +16%, Figure 3A). The same occurred for the reduced CysGly catabolite which increased significantly (*p* < 0.01) in both groups (+45% vs. +24% Figure 3B). Conversely, oxidized Cys (Figure 3C) and CysGly (Figure 3D) declined significantly (*p* < 0.001) in both groups, with similar degrees for Cys (−28% GluReS and −27% GluS), whereas for oxidized CysGly the decrease was greater in GluS compared to GluReS (−37% vs. −30%, respectively).

Table 3 summarizes the data displayed in Figure 1, Figure 2 and Figure 3.

The difference between the t1 levels of reduced glutathione in erythrocytes observed in the two groups, i.e., 1349.87 ± 367.62 in the GluReS group versus 1265.09 ± 144.95 in the GluS group (*p* < 0.013) further supports the more efficient anti-oxidant activity of GluReS compared to GluS.

Since the redox status also has regulatory interactions with the vitamins’ metabolic pathways, we evaluated whether changes occurred in the levels of endogenous vitamins C, A, and E. Noteworthy, endogenous vitamins C, A and E increased significantly in both groups (range *p* < 0.01–0.0001), with the GluReS group showing a much higher increase compared to the GluS group (Table 4). The ∆% of vitamin E refers to the ratio vitamin E:cholesterol.

**Figure 4:**
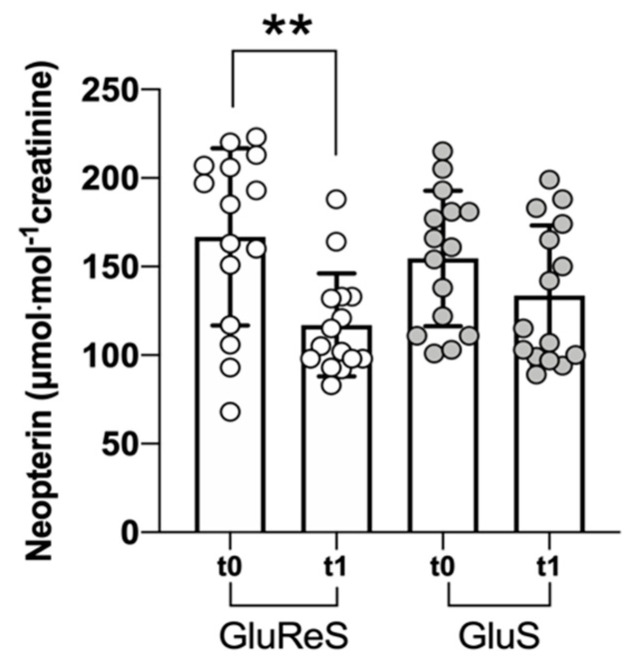
Scatter dot plot of neopterin levels measured in urine before (t0) and after 8 weeks (t1) of dietary supplementation with GluReS or GluS. Data are expressed as the mean ± SD. Changes over time (t1 vs. t0) was significant at ** *p* < 0.01.

Finally, we measured neopterin, an established inflammatory marker [47], in the urine of the participants (Figure 4). Levels of neopterin remained substantially the same in GluS group, whereas it diminished significantly (*p* < 0.01) in the GluReS (−30%).

## 4. Discussion

This study describes a before and after study of dietary intervention with two supplements; the first contains precursors for the endogenous synthesis of glutathione (GluS), whereas the second (GluReS) contains the same precursors in addition to polydatin, a precursor of resveratrol. Ten thiol species, three vitamins and the inflammatory marker neopterin were measured before and after eight weeks of supplementation. Both supplements appeared to improve redox status, as measured by thiol species, with the GluReS performing better overall. Additionally, endogenous vitamin concentrations of vitamins C, E and A were increased with both supplemental protocols, whereas GluReS only induced a significant reduction of neopterin.

**Figure 5:**
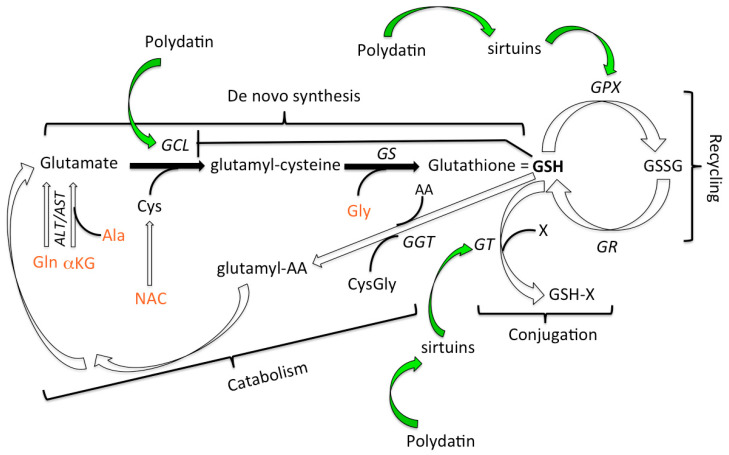
Schematic summary of glutathione synthesis, catabolism, regulation and putative connections with polydatin. Reduced glutathione (GSH) can be produced by continuous recycling mediated by the enzymes glutathione peroxidase (GPX) and glutathione reductase (GR). GPX uses GSH as substrate and oxidizes it to its dimeric oxidized form (GSSG), reducing peroxides and acid peroxides to water and alcohol. GR brings back GSSG to its reduced form GSH which will again be oxidized by GPX, thus leading to the antioxidant status necessary for cellular homeostasis. Instead de novo synthesis of GSH is mediated by glutamate-cysteine ligase (GCL) and glutathione synthetase (GS) when the building blocks (Glutamate, Cys = Cysteine, Gly = Glycine) are available in sufficient quantities. A negative feed-back mechanism exists since GSH inhibits GCL, avoiding unnecessary biosynthesis of GSH. In order to perform its detoxification function GSH is consumed by its conjugation to drugs or other xenobiotics mediated by glutathione S-transferase (GT), whereas its catabolism is initiated by γ-glutamyl transpeptidase (GGT) which yields the CysteinylGlycine (CysGly) dipeptide. Polydatin may impact GSH metabolism either augmenting the quantity or activation of GCL through the Nrf2 pathway or by triggering sirtuins which in turn modulate the activity of GPX and GT. The precursors present in the GluS and GluReS dietary supplements are depicted in red. GSH-X = reduced glutathione bound to a xenobiotic substance; X = xenobiotic substance; AA = aminoacids; NAC = N-acetylcysteine; Ala = alanine; Gln = glutamine; αKG = α-ketoglutarate.

Previous studies demonstrated that the metabolic pathways that lead to the generation of glutathione can be boosted by dietary supplementation with its precursors [18]. To the best of our knowledge however the potential synergy between the increase of sirtuin activity induced by resveratrol, as well as its precursor polydatin, and reduced glutathione (GSH) have not yet been reported. Figure 5 illustrates the pathways leading to GSH synthesis and degradation as well as the points of contact with the activity of polydatin.

The data reported here demonstrate that by providing the selected precursors both dietary supplements (GluReS and GluS) were successful in inducing in erythrocytes a significant increase of endogenous reduced glutathione (GSH) and a concomitant decrease of oxidized glutathione. It should be pointed out that our method measures all oxidized glutathione forms (dimers (GSSG), mixed disulfide forms (GSSR) and protein mixed disulfide forms (ProSSG)), but cannot distinguish among them, which is beyond the scope of our study. All changes in the thiol species occurring after eight weeks of dietary supplementation were highly significant in both arms, ranging from *p* < 0.05 to *p* < 10^−4^, indicating a metabolic acceleration and a boost of intra-cellular (erythrocytes) and extra-cellular (plasma) antioxidant activity.

In both study groups the decrease in erythrocytes of both reduced and oxidized cysteine may indicate that the reduced form is used for the biosynthesis of GSH, decreasing the pool of molecules for oxidation. On the other hand, the plasma levels of oxidized cysteine, which represents a strong pro-oxidant molecule, were significantly decreased, whereas those of reduced cysteine were significantly increased. This increase in plasma can in turn facilitate the transport into the erythrocytes through γ-glutamyl binding. This binding and transport are probably due to the presence of glutamate which is synthesized and made available through the activity of the aminotransferases (ALT and AST) on the substrates α-ketoglutarate and glutamine, both provided by the dietary supplements. In both groups also the catabolite CysGly was affected with concomitant significant reduction of the oxidized form and increase of the reduced form occurring both in erythrocytes and plasma due to the activity of GGT.

Although both study arms behaved in the same manner towards a positive redox outcome from t0 to t1, GluReS was substantially superior to GluS group, in nine out of the 10 thiol species evaluated. The higher performance of GluReS supplementation can be ascribed to the presence of polydatin that provides additional reducing equivalents owing to its antioxidant activity [48] carried out by the hydroxyl group OH in position C4. In addition, polydatin may also act indirectly by activating sirtuins [35,36,37,38], which in turn stimulate phase II detoxifying enzymes and enhance the activity of GST and GPX [41,42,43,44,45,46,47,48,49]. Consumption of reduced glutathione (GSH) molecules through GST activity induces reactivation of GSH de novo synthesis, suggesting that dietary supplement GluReS synergizes with GST through the precursors-mediated biosynthesis of new GSH molecules. While GST is responsible for the detoxifying activity of GSH, the antioxidant action of GSH is carried out by GPX. In our study the reduced and oxidized thiol species demonstrated that GluReS was superior to GluS, suggesting an increased enzymatic activity of GPX induced by polydatin as compared to GR. GluS resulted superior to GluReS only on the reduction of oxidized glutathione at t1, as if the action of GR was delayed. This result could be explained by the fact that GR maintains the same level of activity in the two groups, but in GluReS it is the activity of GPX that appears accelerated due to the presence of polydatin. Finally, polydatin could also activate GLC activity through the NF-E2-related factor 2 (Nrf2) pathway [50] which coordinates the antioxidant and phase II detoxification enzymes to adapt to different stress conditions. Both resveratrol and polydatin have been shown to activate the Nrf2 pathway in different murine models [51,52].

A second important result of our study was the significant increase of endogenous vitamins C, E, and A in both groups at t1. These results can be explained by the fact that the newly synthetized reduced glutathione (GSH) is able to regenerate ascorbic acid (vitamin C) from its precursor dehydroascorbic acid (DHA) through the action of the enzyme dehydroascorbate reductase. Otherwise DHA would irreversibly pass to 2,3-diketogulonic acid, causing a loss of vitamin C. The reduction of DHA by GSH is a very active pathway and leads to almost total recovery of vitamin C. Vitamin C in turn is able to transform the α-tocopherol radical into α-tocopherol (vitamin E), concomitantly neutralizing the free radicals of carotenoids which could have a pro-oxidative effect if not removed by vitamin C. Vitamin E in turn protects from oxidation β-carotene and vitamin A localized intracellularly and in the circulating lipoproteins, thus leading to an increase of vitamin A. GluReS was definitely superior to GluS in upregulating the endogenous levels of vitamins C, E, A, most likely due to the boost of GSH with its effect on the vitamins’ metabolism. Noteworthy, in age-related macular degeneration, vitamins C, E and β-carotene have been shown to be effective in retarding the degenerative process [53] and within the aging population (British participants over 65 years) plasma levels of vitamin C and E have been found to reduce the risk for all-cause mortality, and respiratory mortality, respectively [54].

The significant increase of endogenous vitamins (C, E, A), especially in the polydatin plus glutathione precursors group, is an original and important finding since exogenously provided vitamins may be given in excess to well-nourished people [55] and could possibly be harmful [56]. On the same line, the fact that GSH itself when it has reached the necessary levels establishes a negative feed-back on its de novo synthesis through inhibition of the GCL enzyme [57] implies that providing precursors for endogenous synthesis of GSH could represent a safe, and efficient, strategy.

At last, a significant drop of the inflammatory marker neopterin measured in urine was observed only in the GluReS group. This result can be explained by the anti-inflammatory effect of PD through inhibition of NF-kB activity documented in vitro [58] and in various animal models of inflammation-induced damage of various cells or organs [59,60,61,62] including the kidney [63].

A limitation to this study is represented by the lack of a placebo-controlled arm. Indeed uncontrolled before and after studies have been demonstrated to often be confounded, which leads to an overestimation of the intervention’s effectiveness [64], thus these results, although innovative and significant, require cautious interpretation and further validation.

The age range of our participants was 45–75 years, an important time to implement strategies aiming towards a healthy aging with disease-free years to live. Dietary supplements containing both anti-inflammatory factors such as PD and precursors effective in maintaining GSH levels appear to be a valuable approach. The maintenance of a balanced redox status and of an adequate level of vitamins along with the control of subclinical inflammation can be leveraged for the prevention of chronic degenerative diseases associated with aging. Depletion of cellular GSH induces ferroptosis [65], an iron-dependent non-apoptotic form of cell death [66] and low levels of GSH are found in HIV-infected individuals [67] and in a number of age-related diseases [57], such as cataract [68], Alzheimer’s [69], diabetes and cancer [70]. Finally, a recent paper by Lian and colleagues reported that GSH de novo synthesis, but not GSH recycling, is required for activation and proliferation of murine T lymphocytes [71], underlying the importance of providing GSH precursors to maintain an immune-competent status, and providing an additional potential application of GluS and/or GluReS supplements in the prevention of infectious diseases.

## 5. Conclusions

This study provides preliminary but strong evidence for the use of GSH precursors combined with polydatin to maintain the redox status into an ideal range while at the same time activating sirtuins which, in addition to their synergistic effects on the redox status, exert a multitude of beneficial effects on cellular metabolism and immune functions. These results should encourage further testing of GluReS in randomized placebo-controlled studies on the prevention of age-associated diseases and the optimization of immunocompetency.

## Figures and Tables

**Table 1:** Anthropometric data of the two groups.

	GluReS	GluS
Weight (kg)	68.65 (14.61)	75.36 (13.24)
Height (m)	1.69 (0.11)	1.70 (0.09)
BMI (kg/m^2^)	23.80 (2.45)	25.87 (3.62)
Waistline (cm)	90.54 (8.05)	98.83 (11.95)
Heart rate	68.46 (9.39)	65.08 (8.55)
Sistolic blood pressure	122.92 (10.77)	125.58 (12.72)
Diastolic blood pressure	86.77 (4.73)	83.08 (8.71)

GluReS = Glutathione and Resveratrol Synthesis group; GluS = Glutathione Synthesis group. Values represent mean (±SD). BMI = body mass index.

**Table 2:** The formulation of the two dietary supplements.

	GluReS	GluS
	mg	mg
glutamine, α-ketoglutarate	217	217
*N*-acetylcysteine	210	210
glycine	105	105
alanine	126	126
sodium selenite	7	7
polydatin	35	---
total	700	665

**Table 3:** Before and after concentrations (µM) of reduced and oxidized forms of glutathione, cysteine and cysteinylglycine in erythrocytes and plasma for the two groups.

Erythrocytes	GluReS			GluS		
	t0	t1	%∆	t0	t1	%∆
Glutathione						
reduced	967.59 ± 283.23	1349.87 ± 367.62	+40	958.42 ± 123.74	1265.09 ± 144.95	+32
oxidized	1262.95 ± 321.77	560.47 ± 251.47	−56	1115.35 ± 346.66	231.67 ± 196.89	−79
Cysteine						
reduced	17.51 ± 2.17	13.74 ± 3.24	−22	17.94 ± 1.77	14.49 ± 1.26	−19
oxidized	28.46 ± 3.04	18.72 ± 1.72	−34	27.24 ± 3.30	20.77 ± 2.89	−24
Cysteinylglycine						
reduced	5.43 ± 1.81	7.14 ± 2.26	+32	3.81 ± 0.51	4.77 ± 0.63	+25
oxidized	3.45 ± 0.67	1.92 ± 0.37	−44	3.40 ± 0.70	1.79 ± 0.28	−47
**Plasma**	**GluReS**			**GluS**		
	**t0**	**t1**	**%∆**	**t0**	**t1**	**%∆**
Cysteine						
reduced	14.59 ± 1.67	20.73 ± 5.05	+42	16.71 ± 1.36	19.35 ± 2.80	+16
oxidized	300.81 ± 75.58	215.48 ± 46.55	−28	292.28 ± 34.31	213.65 ± 39.17	−27
Cysteinylglycine						
reduced	5.63 ± 0.83	8.19 ± 2.30	+45	7.62 ± 2.06	9.45 ± 2.13	+24
oxidized	77.55 ± 25.95	53.95 ± 12.50	−30	74.99 ± 10.48	47.28 ± 10.72	−37

**Table 4:** Summary of the change from t0 to t1 (∆%) and significance (*p*) of the three vitamins for the two groups.

	vitamin C	*p*	vitamin A	*p*	vitamin E	*p*
	∆%		∆%		∆%	
GluReS	+37	<0.01	+33	<0.01	+58	<0.0001
GluS	+11	<0.001	+14	<0.001	+39	<0.0008
